# Slow releasing sulphide donor GYY4137 protects mice against ventilator-induced lung injury

**DOI:** 10.1186/s40635-025-00753-9

**Published:** 2025-04-22

**Authors:** Lilly Veskemaa, Mahdi Taher, Jan Adriaan Graw, Adrian Gonzalez-Lopez, Roland C. E. Francis

**Affiliations:** 1 Department of Anaesthesiology and Intensive Care Medicine CCM/CVK, Charité-Universitätsmedizin Berlin, Corporate Member of Freie Universität Berlin and Humboldt Universität zu Berlin, Berlin, Germany; 2 Department of Anaesthesiology, Friedrich-Alexander-Universität Erlangen-Nürnberg (FAU), Universitätsklinikum Erlangen, Krankenhausstraße 12, 91054 Erlangen, Germany; 3 Centro de Investigación Biomédica en Red-Enfermedades Respiratorias, Instituto de Salud Carlos III, C/Monforte de Lemos 3-5, Pabellón 11, Planta 0, 28029 Madrid, Spain; 4 Department of Anaesthesiology and Intensive Care Medicine, Universitätsklinikum Ulm, Ulm University, 89081 Ulm, Germany

**Keywords:** Ventilator-induced lung injury, Biotrauma, Sulphide donors, Hydrogen sulphide

## Abstract

**Background:**

Cyclic stretching of the lung during mechanical ventilation induces inflammation that contributes to the development of ventilator induced lung injury. Hydrogen sulphide (H_2_S) is an endogenous gasotransmitter known for its anti-inflammatory properties. However, the administration of exogenous H_2_S is constrained by its narrow therapeutic window, rapidly leading to potentially toxic peak concentrations. Alternatively, slow-release sulphide donors, such as GYY4137, offer a more controlled delivery. The primary aim of this study is to assess the efficacy and safety of GYY4137 in mitigating VILI.

**Methods:**

Anaesthetised male C57BL/6 J mice were pretreated with an intraperitoneal injection of GYY4137 (50 mg/kg, *n* = 14) or an equivalent volume of phosphate-buffered saline (controls, *n* = 13) and were then subjected to high tidal volume ventilation (*V*_T_ 40–42.5 ml/kg) for a maximum of 4 h.

**Results:**

GYY4137 pretreatment led to a notable 50% increase in survival rates compared to controls (*p* = 0.0025). It also improved arterial oxygenation after high V_T_ ventilation, with arterial partial pressure of oxygen (PaO2) of 64 mmHg (IQR 49–125 mmHg) vs. 44 mmHg (IQR 42–51 mmHg) in controls (*p* < 0.001). Additionally, GYY4137 reduced total protein concentration in bronchoalveolar lavage fluid by 30% (*p* = 0.024) and lowered IL-1β levels by 40% (*p* = 0.006). GYY4137 mitigated the decline in dynamic respiratory system compliance caused by high V_T_ ventilation, showing values of 24 μl/cmH_2_O (IQR 22–27) compared to 22 μl/cmH_2_O (IQR 22–24) in controls (*p* = 0.017). GYY4137 had minimal effects on antioxidant gene expression related to the erythroid nuclear factor 2, and it did not affect glutathione metabolism, the nuclear factor kappa B pathway, or the endoplasmic reticulum stress response.

**Conclusions:**

In this mouse model of VILI, pretreatment with GYY4137 showed protective effects. GYY4137 significantly improved survival. It also improved arterial blood oxygenation and dynamic respiratory system compliance, and mitigated the development of lung oedema and inflammation.

## Introduction

Mechanical ventilation has a central role in the management of respiratory failure. However, the unphysiological positive pressure changes applied onto the lung tissue can cause ventilator-induced lung injury (VILI). The pathophysiological mechanisms involved in the development of VILI include tidal recruitment (atelectrauma) and over-distension (baro- and volutrauma). These mechanical forces can also induce the development of biotrauma, characterised by inflammation and oxidative stress caused by cyclic stretching of the lung tissue [1]. This can lead to the formation of lung oedema and worsening of lung function. Notably, biotrauma can contribute to systemic inflammation and oxidative stress, leading to multiple-organ failure [2].

Hydrogen sulphide (H_2_S) is an endogenous gasotransmitter involved in the regulation of immune responses [3]. Down-regulation of endogenous H_2_S production is associated with development and progression of autoimmune disease. Thus, H_2_S has a therapeutic potential in diseases that involve excessive inflammation.

Exogenous H_2_S inhalation provides protection against lipopolysaccharide (LPS)-induced lung injury. In a mouse model, inhalation of 80 ppm H_2_S reduced inflammation and oxidative stress in the lungs following intranasal administration of LPS [4]. Similarly, in a mouse model of VILI using moderate tidal volumes, inhalation of 80 ppm H_2_S provided protection against lung injury [5]. In contrast, in a mouse model of VILI using very high-tidal volumes, inhalation of 60 ppm H_2_S exacerbated the lung injury [6]. However, in the same study intra-peritoneal sodium sulphide reduced inflammation and oxidative stress in the lungs caused by high tidal volume ventilation and provided protection against VILI.

As a drug, H_2_S has a very narrow therapeutic window when administered as inhaled gas or in the form of fast-releasing sulphide donors such as sulphide salts. Slow-releasing sulphide donors offer a promising alternative as they avoid concentration spikes and thus are less toxic. GYY4137 (morpholin-4-ium 4 methoxyphenyl(morpholino) phosphonodithioate) is a water-soluble, slow-releasing H_2_S donor that mimics the slow physiological production of H_2_S [7].

Similar to H_2_S inhalation, GYY4137 has been shown to protect against LPS-induced lung injury and reduce airway inflammation [8, 9]. In addition, two studies showed its beneficial anti-inflammatory properties in mice infected with respiratory syncytial virus infection [10, 11]. GYY4137 also reduced intrapulmonary inflammation and acute lung injury after infrarenal aortic cross-clamping [12].

The exact molecular mechanism by which exogenous sulphide species exert this anti-inflammatory and antioxidant effect remains unclear. Previous studies have shown an association with the inhibition of the nuclear factor kappa B (NFκB) pathway as well as the up-regulation of nuclear erythroid 2-related factor 2 (Nrf2) dependent antioxidant gene expression [6, 11, 13, 14]. More recently, it was shown that cyclic stretching of lung cells induces endoplasmic reticulum stress that was inhibited with sulphide donor sodium hydro-sulphide [15].

Based on these considerations, we hypothesised that GYY4137 would reduce mechanical ventilation-induced inflammation and thus provide protection against VILI. In this study we tested whether pretreatment with GYY4137 prior to lethal high V_T_ ventilation improved survival (primary outcome measure). The secondary outcome measures were arterial blood oxygenation, respiratory system compliance, lunge oedema and pulmonary inflammation. We further explored possible mechanisms of the protective effect of GYY4137.

## Methods

The study was approved by the Institutional Animal Care and Use Committee (Tierversuchskommision Landesamt für Gesundheit und Soziales, Berlin, Germany, approval number G0189/15). Male C57BL/6 J mice, 8–10 weeks, were obtained from Charles River Laboratories in Germany and maintained under standard laboratory conditions provided by the Animal Facility of the Charité—Universitätsmedizin Berlin (FEM—Forschungseinrichtung für experimentelle Medizin, Tierhaltung). Adherence to the ARRIVE guidelines was observed where applicable (https://arriveguidelines.org/resources/author-checklists).

### Mouse model of VILI (workflow diagram)

Mice (body weight 24.0 ± 0.8 g, mean ± SD) were anaesthetised with an intra-peritoneal injection (i.p.) of ketamine (120 mg/kg) and xylazine (6 mg/kg). A surgical tracheostomy was performed, and mice were ventilated in a volume-controlled mode at a tidal volume (*V*_T_) of 10 ml/kg, respiratory rate of 90 breaths/min, positive end-expiratory pressure (PEEP) of 2 cmH_2_O, and fraction of inspired oxygen (F_I_O_2_) of 0.4 for 1 h (baseline ventilation). The common carotid artery was catheterised for the purpose of blood pressure monitoring, continuous infusion of maintenance anaesthesia, and blood sampling.
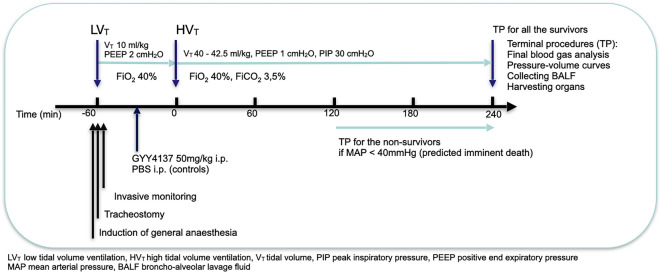


One hour after the initiation of baseline ventilation, high tidal volume (HV_T_) ventilation was initiated—V_T_ was increased to 40–42.5 ml/kg to reach a peak inspiratory airway pressure of 30 cmH_2_O. PEEP was set to 1 cmH_2_O in order to induce high stretch mechanical ventilation with sufficient stress and strain to cause mortality within 4 h of ventilation. Furthermore, the respiratory rate was reduced to 60 breaths/min and 3.5% of CO_2_ was added to the inhaled gas (40% oxygen concentration) to prevent respiratory alkalosis. The ventilation was continued for a maximum duration of 4 h of HV_T_ ventilation or until the mean arterial pressure dropped below 40 mmHg for more than 10 s, at which point imminent circulatory arrest could be predicted in this mouse model, as other authors have reported [16]. Alveolar recruitment manoeuvres were performed at 30-min intervals during baseline ventilation and at 60-min intervals during HV_T_ ventilation. Body temperature was maintained at approximately 37.5 °C using a heating pad thermostat.

### Experimental groups

C57BL/6 J mice were randomly assigned to receive a single i.p. injection of 50 mg/kg GYY4137 (Cayman Chemical, CAS Registry Number 106740–09-4, 17 ml/kg *n* = 14), or Dulbecco’s phosphate buffered saline (controls, Gibco™, Thermo Fischer Scientific, USA, *n* = 13). GYY4137 was dissolved in PBS, yielding a concentration of 3 mg/ml (maximum solubility according to product information). It was divided into small aliquots and stored at − 80 °C. The dose and the timing of GYY4137 were based on previously published pharmacological characterisation of GYY4137 and studies conducted on mice [8, 12, 17]. This approach adhered to the 3R principle (replace, reduce, refine) in animal research by omitting additional dose–response studies.

To ensure that both groups—GYY4137 and controls—received the same amount of fluids during the experiments, the GYY4137 group received only one extra fluid bolus during low tidal volume ventilation and the control group received two boluses, resulting in a total fluid bolus amount of 28 ml/kg for both groups.

Another group of mice was pretreated with PBS (10 ml/kg) and subjected to low tidal volume ventilation only (LV_T_). These mice were handled identically to HV_T_ groups, with the only difference being that the mechanical ventilation was maintained at baseline level (10 ml/kg) for 5 h.

To assess the effect of GYY4137 at baseline, additional groups of mice were sacrificed 1 h after pretreatment with either PBS or GYY4137, without undergoing HV_T_ ventilation.

Randomisation of animals followed the principles of simple randomisation. Blinding was not possible as all experiments and analyses were performed by the same person.

### Assessment of respiratory system mechanics and blood gas analysis

V_T_, peak inspiratory airway pressure and PEEP were measured continuously during the experiment. Pressure volume curves were recorded before initiation of HV_T_ ventilation and at the end of each experiment. Inspiratory capacity and compliance of the respiratory system before and after HV_T_ ventilation were calculated from the recorded data. Arterial oxygen partial pressures were measured once at the end of the experiment as part of the terminal procedures. All details are available in the Supplemental Digital Content.

### Assessment of protein and pro-inflammatory cytokines in bronchoalveolar lavage fluid (BALF)

Total protein concentration in BALF was measured using the bicinchoninic acid assay (Thermo Scientific™ Pierce™ BCA™ Protein-Assay, USA) according to the manufacturer’s instructions, to assess pulmonary oedema formation.

The pro-inflammatory cytokine interleukin-1 beta (IL-1β) was measured using uncoated ELISA kits (Invitrogen by Thermo Fischer Scientific, USA) according to the manufacturer’s instructions.

### Assessment of the inflammatory pathway of NFκB

Phosphorylated p65 and total p65 as well as p105 and p50 were determined by Western blot analyses using specific antibodies (Cell Signaling, USA) to assess the activation of the canonical pathway of NFκB.

### Assessment of oxidative stress and pulmonary gene expression

Reduced (GSH), oxidised (GSSG) and total (GSH + GSSG) glutathione concentrations in lung tissue were measured using an enzymatic assay (Cayman Chemical, USA) according to the manufacturer’s instructions.

Quantitative real-time polymerase chain reaction was used to determine the relative gene expression of pulmonary nuclear factor (erythroid-derived 2)-like 2 (*Nrf2*), NAD(P)H quinone oxidoreductase (*Nqo1*), superoxide dismutase 1 (*Sod1*), superoxide dismutase 2 (*Sod2*), glutathione peroxidase 2 (*Gpx2*) and heme oxygenase 1 (*Hmox1*). Further detailed descriptions of the biochemical and molecular biological procedures are available in the Supplemental Digital Content.

### Assessment of endoplasmic reticulum stress response activation

Quantitative real-time polymerase chain reaction was used to determine the relative gene expression of pulmonary activating transcription factor 4 (*Atf4*), activating transcription factor 6 (*Atf6*) and x-box binding protein 1 (*Xbp1*). A more detailed description of the biochemical and molecular biological procedures is available in the Supplemental Digital Content.

### Statistical analysis

A convenience sample size was used based on our previous experience in this lethal VILI model and with survival as the primary outcome [6]. Therefore, a minimum of 10 per group was targeted for controls and interventions. Due to the small sample size most groups failed to pass the Shapiro–Wilk test for normality, thus the Mann–Whitney *U* test was used for non-parametric comparison of two groups. Survival curves were compared using the log-rank Mantel–Cox test.

All tests were performed using GraphPad Prism® version 10.1.1 for MacBook (GraphPad Software, San Diego, CA, USA). Data are presented as median and interquartile range (IQR). Data representing relative changes, such as relative gene expression and Western blot analysis, are expressed as mean ± SD (standard deviation). A *p* value less than 0.05 was considered statistically significant.

## Results

### GYY4137 improves survival in mice subjected to HV_T_ ventilation

**Fig. 1:**
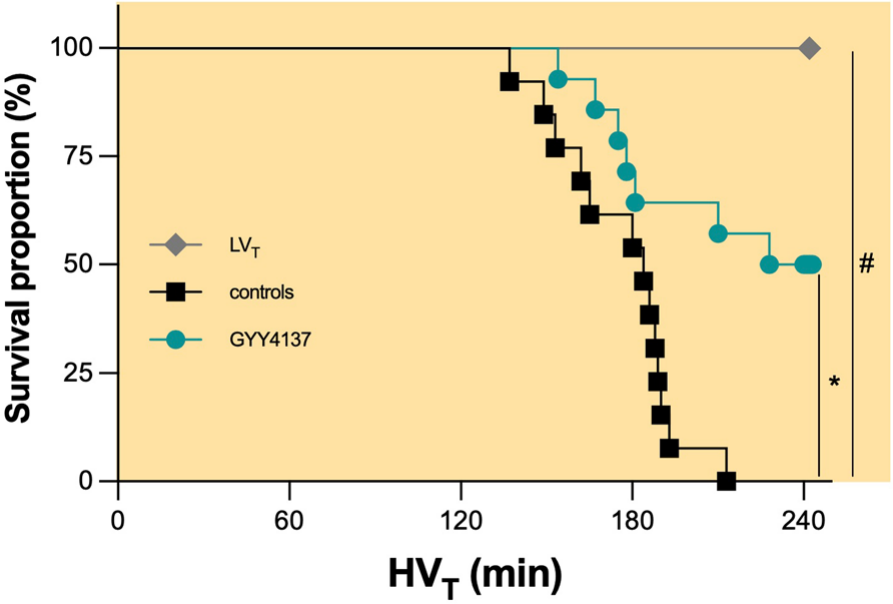
Survival. Groups: controls (*n* = 13)—pretreatment with PBS and HV_T_ ventilation, GYY4137 (*n* = 14)—pretreatment with GYY4137 and HV_T_ ventilation, LV_T_, (*n* = 7)—pretreatment with PBS and LV_T_ ventilation. *p = 0.0025 GYY4137 vs. controls, #*p* < 0.001 LV_T_ vs. HV_T_ (log-rank Mantel–Cox test)

HV_T_ ventilation caused 100% mortality within 4 h among controls (Fig. 1). Pretreatment with GYY4137 resulted in a 50% survival rate at 4 h (Fig. 1). The median survival time was 184 min in controls vs. 236 min in mice pretreated with GYY4137 (*p* = 0.0025).

### GYY4137 improves arterial oxygenation in mice subjected to HV_T_ ventilation

**Fig. 2:**
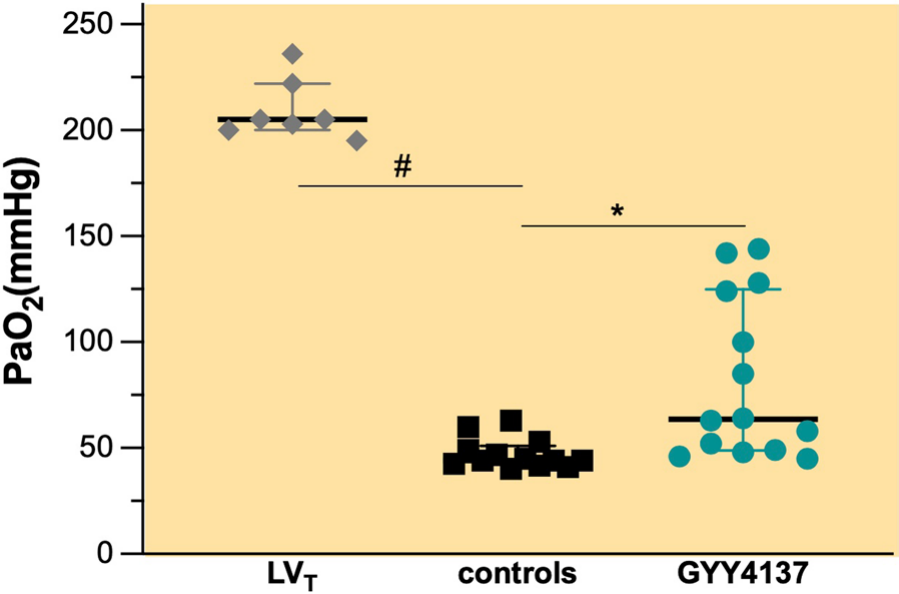
Arterial blood oxygen (PaO_2_) tension. Groups: controls (*n* = 13)—pretreatment with PBS and HV_T_ ventilation, GYY4137 (*n* = 14)—pretreatment with GYY4137 and HV_T_ ventilation, LV_T_, (*n* = 7)—pretreatment with PBS and LV_T_ ventilation. The inspired oxygen fraction was 0.4 in all groups. All values are depicted as median and IQR. *p < 0.001 GYY4137 vs. controls (Mann–Whitney *U* test). #*p* < 0.001 LV_T_ vs. HV_T_ (Mann–Whitney *U* test)

All mice subjected to LV_T_ ventilation survived mechanical ventilation and had a normal arterial oxygenation with PaO_2_ 205 mmHg (Fig. 2, IQR 200–222 mmHg, F_I_O_2_ 0.4) at the end of the experiment. In contrast, HV_T_ ventilation severely impaired arterial oxygenation, but mice pretreated with GYY4137 showed an improved arterial oxygenation at the end of HV_T_ ventilation compared with controls (Fig. 2, PaO_2_ 64 mmHg, IQR 49–125 mmHg vs. 44 mmHg, IQR 42–51 mmHg, *p* < 0.00, F_I_O_2_ 0.4).

### GYY4137 attenuates the development of lung oedema and improves respiratory system compliance in mice subjected to HV_T_ ventilation

**Fig. 3:**
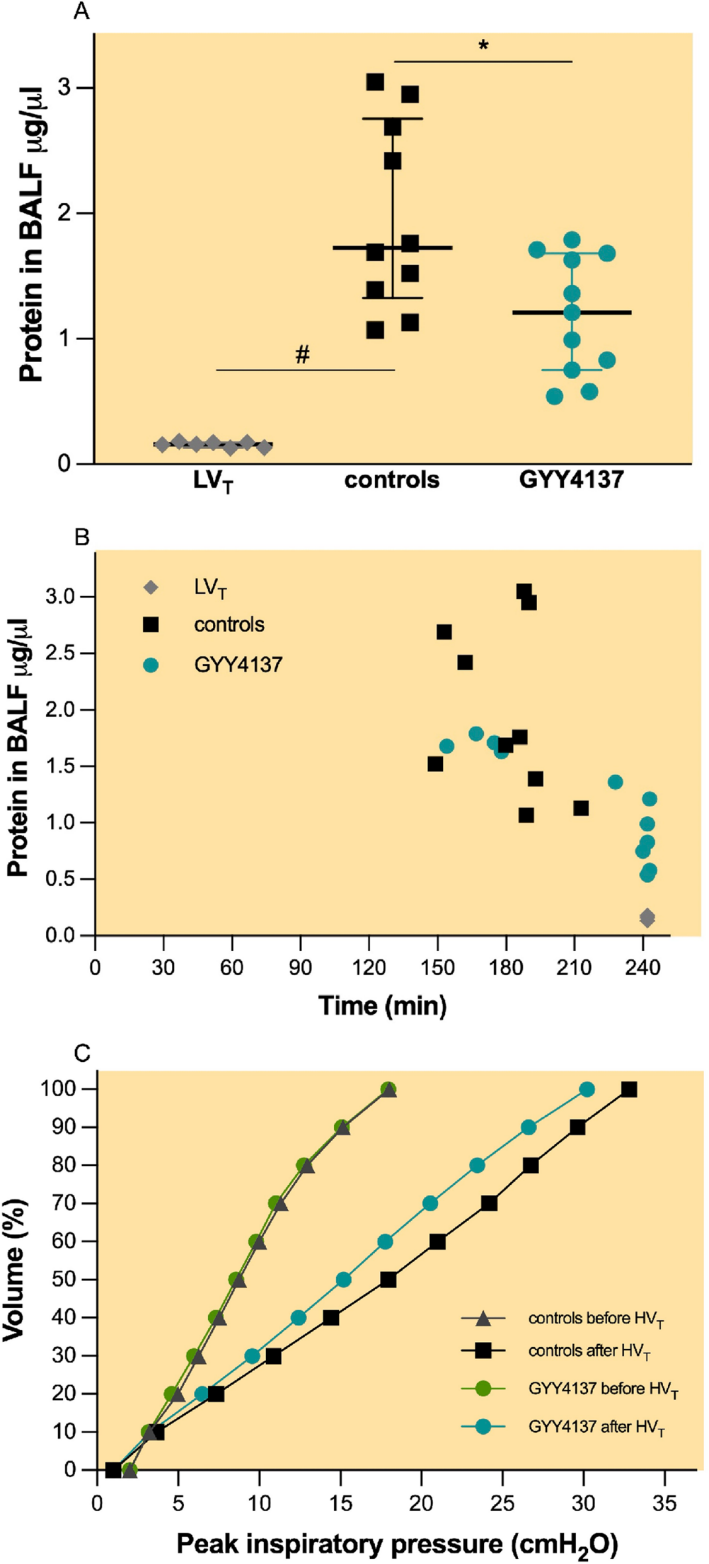
Oedema formation and respiratory system mechanics. **A** Total protein concentration in BALF; groups: controls (*n* = 10)—pretreatment with PBS and HV_T_ ventilation, GYY4137 (*n* = 11)—pretreatment with GYY4137 and HV_T_ ventilation, LV_T_, (*n* = 7)—pretreatment with PBS and LV_T_ ventilation. All values are depicted as median and IQR. # *p* < 0.001 LV_T_ vs. HV_T_ (Mann–Whitney *U* test). **B** Scatter plot depicting total concentration of protein in BALF vs. duration of mechanical ventilation. Groups: same as graph A. **C** Pressure–volume curves before and after HV_T_ ventilation. Groups: controls (*n* = 13), GYY4137 (*n* = 14). Pressure–volume curves are displayed as the mean of all curves in each experimental group. For clarity, only the mean values are displayed

Total protein concentration in BALF—a surrogate for the degree of pulmonary oedema—was low in mice subjected to LV_T_ ventilation (Fig. 3A, 0.2 μg/μl, IQR 0.1–0.2 μg/μl). Compared with LV_T_ ventilation, HV_T_ ventilation caused an 8.5-fold increase in BALF protein concentration in controls (Fig. 3A, 1.7 μg/μl, IQR 1.3–2.8 μg/μl, *p* < 0.001). Pretreatment with GYY4137 reduced the total protein concentration in BALF after HV_T_ ventilation by 30% (Fig. 3A, 1.2 μg/μl, IQR 0.8–1.7 μg/μl, *p* = 0.024) compared with controls.

Analysis of potential correlations between the BALF total protein concentration and the duration of HV_T_ ventilation showed a strong negative correlation in the GYY4137 group (Fig. 3B, r = − 0.8, *p* < 0.001) and in the pooled sample of both groups (Fig. 3B, r = − 0.7, *p* = 0.008), but not in the control group (Fig. 3B, r = − 0.3, *p* = 0.4).

Dynamic and semi-static respiratory system compliance was comparable between groups at baseline (Table 1). In controls, dynamic compliance decreased by approximately 45% (*p* < 0.001) and semi-static compliance by 49% (*p* < 0.001) after HV_T_ ventilation (Table 1). The reduction of respiratory system compliance was also illustrated by a rightward shift of the pressure–volume curve after HV_T_ ventilation (Fig. 3C, black line).

Pretreatment with GYY4137 reduced the HV_T_ ventilation-associated decrease in dynamic respiratory system compliance (Table 1, *p* = 0.017) and attenuated the rightward shift of the pressure–volume curve after HV_T_ ventilation (Fig. 3C, turquoise line).

**Table 1:** Respiratory system mechanics

	Dynamic respiratory compliance (μl/cm H_2_O)		Semistatic respiratory compliance (μl/cm H_2_O))	
	Before HV_T_	After HV_T_	Before HV_T_	After HV_T_
Controls	40, IQR = 36–41	22^#^, IQR = 22–24	59, IQR = 55–61	30^#^, IQR= 28–30
GYY4137	38, IQR = 36–40	24*^#^, IQR = 22–27	58, IQR = 57–60	31^#^, IQR= 28–36

Respiratory system mechanics measured before the initiation of high tidal volume ventilation (before HV_T_) and after HV_T_ ventilation (after HV_T_) pretreated with phosphate buffered saline (controls, n=12) or GYY4137 (n=14). All values presented median and IQR. *p<0.05 GYY4137 versus controls after HV_T_(Mann-Whitney U-test). #p<0.05 Before HV_T_ vs After HV_T_ (Mann-Whitney U-test). Missing samples: 1 control in semi-static compliance due to technical failure to record pressure-volume curves

### GYY4137 ameliorates the pro-inflammatory response in the lung subjected to HV_T_ ventilation independently of NFκB pathway

**Fig. 4:**
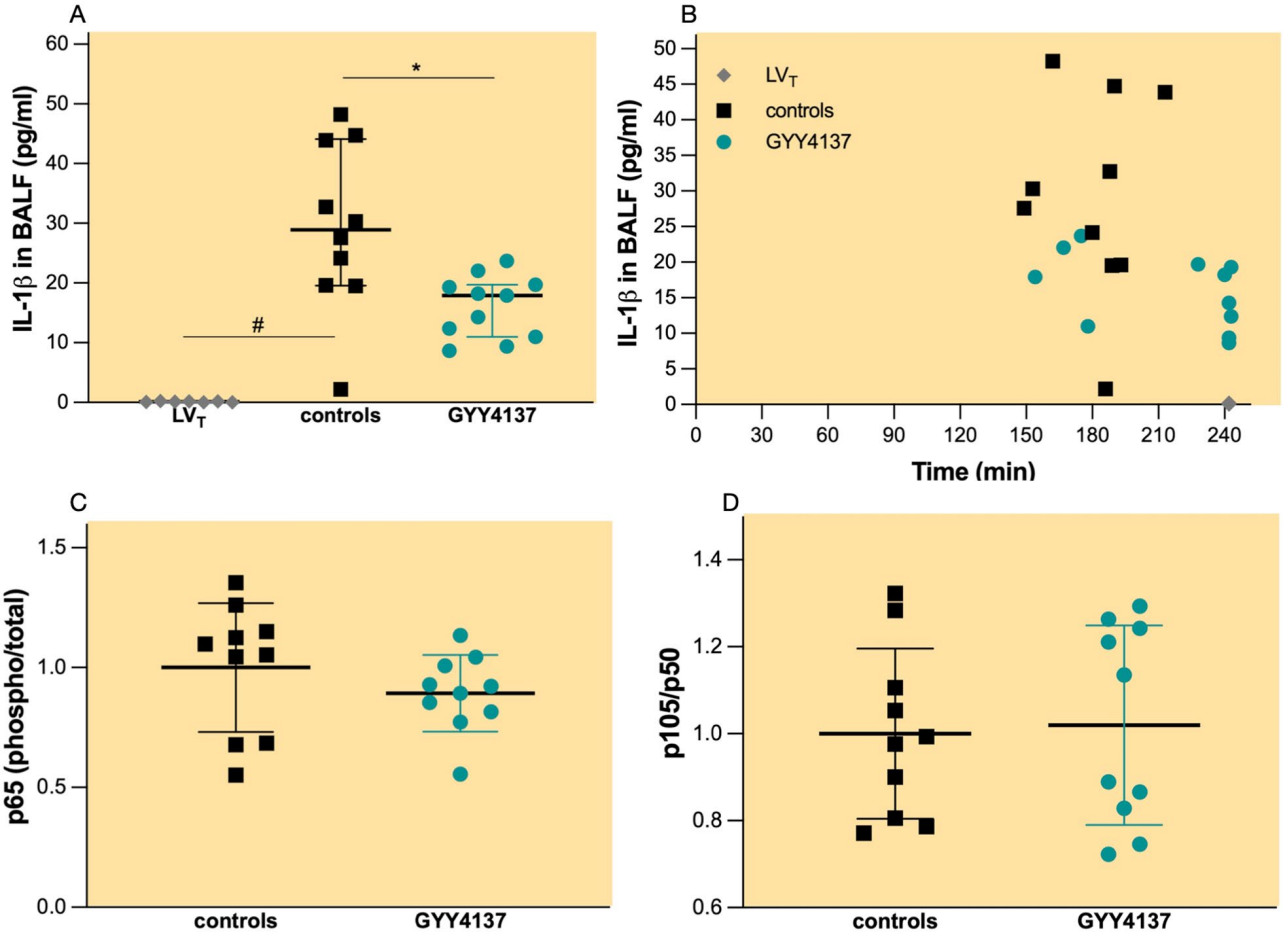
Inflammation in the lung.** A** concentration of IL–1β in BALF; groups: controls (*n* = 10)—pretreatment with PBS and HV_T_ ventilation, GYY4137 (*n* = 11)—pretreatment with GYY4137 and HV_T_ ventilation, LV_T_, (*n* = 7)—pretreatment with PBS and LV_T_ ventilation. All values are depicted as median and IQR. *p < 0.05 GYY4137 vs. controls (Mann–Whitney *U* test). #*p* < 0.05 LV_T_ vs. HV_T_ (Mann–Whitney *U* test). **B** Scatter plot depicting concentrations of IL-1β in BALF vs. duration of mechanical ventilation. **C** phosphorylated p65 and total p65 ratio and (**D**) p105 and p50 ratio in lung tissue after HV_T_ ventilation; groups: controls (*n* = 10), GYY4137 (*n* = 10). Protein concentrations are expressed as fold change relative to the average normalised concentration in no HV_T_ controls (mean ± SD). *p < 0.05 GYY4137 vs. controls (Mann–Whitney *U* test). #*p* < 0.05 LV_T_ vs. HV_T_ (Mann–Whitney *U* test)

In mice subjected to LV_T_ ventilation, BALF IL–1β concentrations (Fig. 4A, 0.1 pg/ml, IQR 0.02–0.2 pg/ml) remained at very low levels. After HV_T_ ventilation, 29 pg/ml (IQR 20–44 pg/ml) of IL–1β was detected in BALF (Fig. 4A, p < 0.001 controls vs. HV_T_). Pretreatment with GYY4137 resulted in a 40% reduction of IL–1β in BALF after HV_T_ ventilation (Fig. 4A, 18 pg/ml, IQR 11–20, *p* = 0.006). BALF IL–1β concentrations did not correlate with the duration of HV_T_ ventilation (Fig. 4B).

Controls and pretreatment with GYY4137 resulted in comparable phosphorylated p65/total p65 (Fig. 4C) and p105/p50 ratio (Fig. 4D) after HV_T_ ventilation.

### GYY4137 has minimal effect on pulmonary antioxidant Nrf2-dependent gene expression

To assess the lung antioxidant capacity at baseline, we measured the Nrf2-dependent gene expression in the lungs of mice sacrificed 1 h after pretreatment and not subjected to HV_T_ ventilation.

**Fig. 5:**
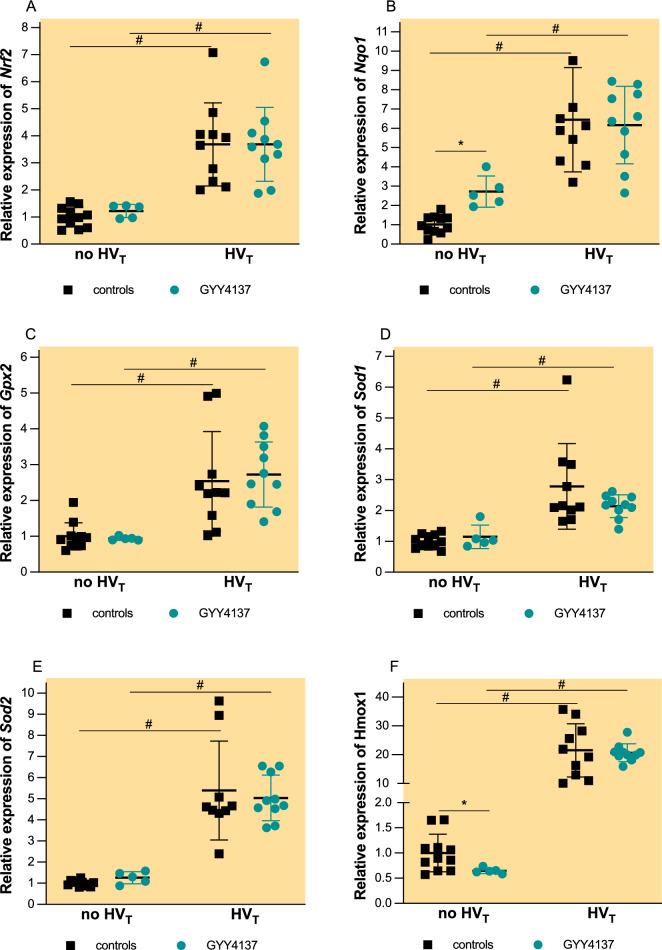
Pulmonary messenger RNA (mRNA) concentrations of *Nrf2* (**A**), *Nqo1* (**B**), *Gpx2* (**C**), *Sod1* (**D**), *Sod2* (**E**), and *Hmox1* (**D**). Groups: controls no HV_T_ (*n* = 11)—pretreatment with PBS and no mechanical ventilation, GYY4137 no HV_T_ (*n* = 5)—pretreatment with GYY4137 an no mechanical ventilation, controls HV_T_ (*n* = 10) pretreatment with PBS and HV_T_ ventilation, GYY4137 HV_T_ (*n* = 10)—pretreatment with GYY4137 and HV_T_ ventilation. mRNA concentrations are expressed as fold change relative to the average expression values in no HV_T_ controls (mean ± SD). *p < 0.05 vs. controls among no HV_T_ (Mann–Whitney *U* test), #*p* < 0.05 no HV_T_ vs. HV_T_ (Mann–Whitney *U* test)

Pretreatment with GYY4137 resulted in a 2.5-fold increase in the relative expression of pulmonary messenger RNA (mRNA) *of Nqo1* (Fig. 5B, p < 0.001) and a 1.6-fold decrease in *Hmox1* (Fig. 5F, p = 0.038) compared with controls at baseline (no HVT). Pretreatment with GYY4137 did not affect the pulmonary expression of *Nrf2* (Fig. 5A), *Gpx2* (Fig. 5C), *Sod1* (Fig. 5D) and *Sod2* (Fig. 5E) at baseline.

HV_T_ ventilation induced a 6.4-fold increase in *Nqo1* (Fig. 5B, p < 0.001) and *21.5*-fold increase in *Hmox1* (Fig. 5F, p < 0.001) expression as well as 3.7-fold increase in *Nrf2* (Fig. 5A, p < 0.001), 2.5-increase in *Gpx2* (Fig. 5C, p < 0.001), 2.7-fold increase in Sod1 (Fig. 5D, p < 0.001), and 6.9-fold increase in *Sod2* (Fig. 5E, p < 0.001). Pretreatment with GYY4137 did not alter the pulmonary expression of *Nrf2, Nqo1, Gpx2, Sod1, Sod2, Hmox1* after HV_T_ ventilation (Fig. 5)*.*

**Fig. 6:**
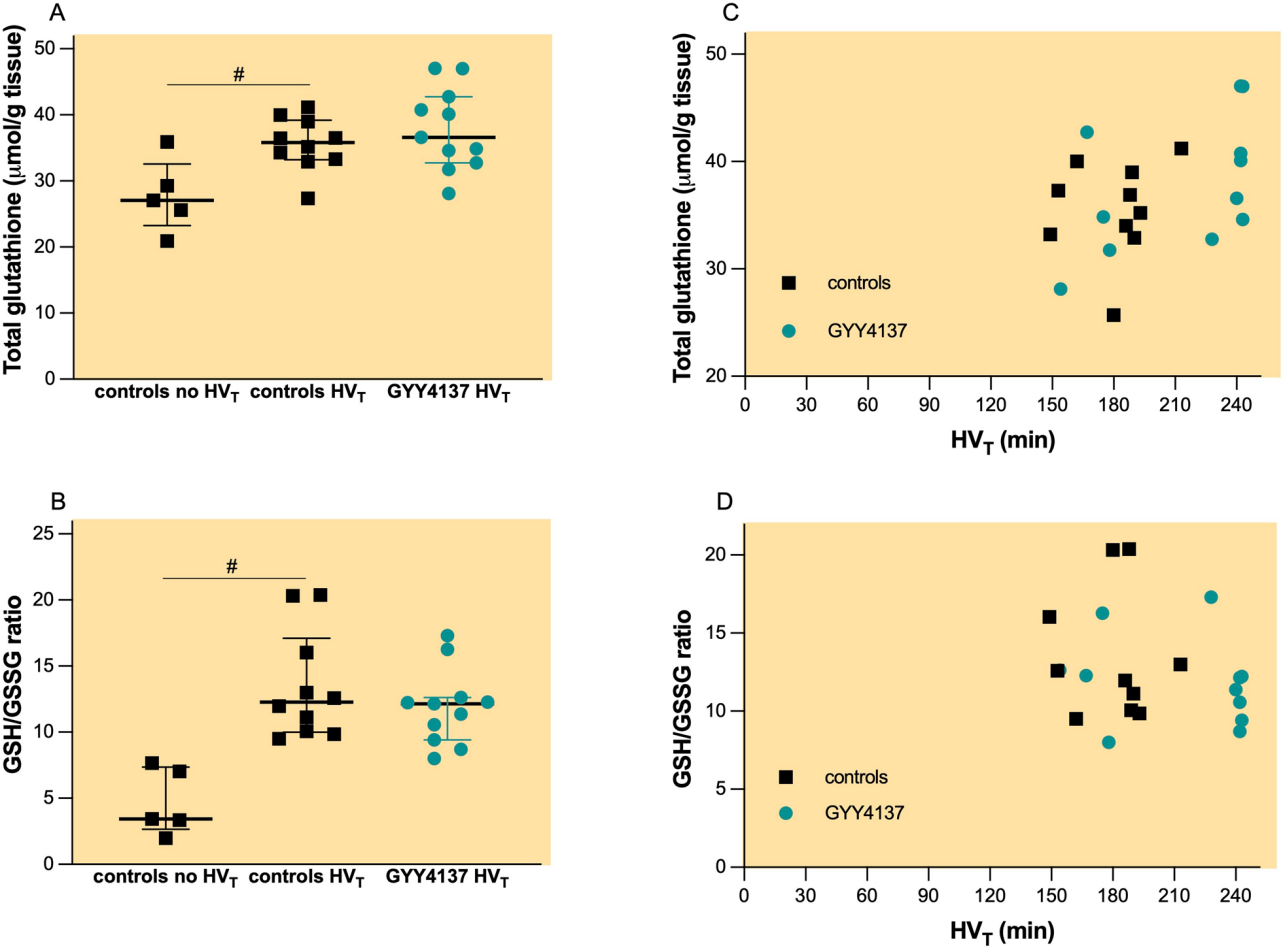
Total glutathione (**A**) and reduced (GSH) to oxidized glutathione (GSSG) ratio (**B**). Groups: controls no HV_T_ (*n* = 5)—pretreatment with PBS and no mechanical ventilation, controls HV_T_ (*n* = 10) pretreatment with PBS and HV_T_ ventilation, GYY4137 HV_T_ (*n* = 11)—pretreatment with GYY4137 and HV_T_ ventilation. All values are depicted as median and IQR. #*p* < 0.05 no HV_T_ vs. HV_T_ (Mann–Whitney *U* test)

HV_T_ ventilation also increased the total glutathione concentration (Fig. 6A, 36 µmol/g tissue, IQR = 33–39 µmol/g vs. 27 µmol/g, IQR 23–33 µmol/g, *p* = 0.019) and GSH/GSSG ratio (Fig. 6B, 12.3, IQR 10–17 vs. 3.4, IQR = 2.7–7.4, *p* < 0.001). Pretreatment with GYY4137 had not effect on total glutathione concentration (Fig. 6A, 37 µmol/g, IQR 33–43 µmol/g, *p* = 0.467) or GSH/GSSG ratio (Fig. 6B, 12.2, IQR = 9.4–12.6, *p* = 0.467). Analysis of potential correlations between lung total glutathione concentration or GSH/GSSH and the duration of HV_T_ ventilation showed a positive correlation for total glutathione (Fig. 6C, r = 0.4594, *p* = 0.036) but not for GSH/GSSG (Fig. 6D).

### GYY4137 does not affect expression of genes downstream of endoplasmic reticulum stress response

At baseline, in mice pretreated with GYY4137 but not exposed to HV_T_ ventilation, one mouse was detected as a significant outlier in all three gene expressions. This mouse was excluded from the analysis (data not shown).

**Fig. 7:**
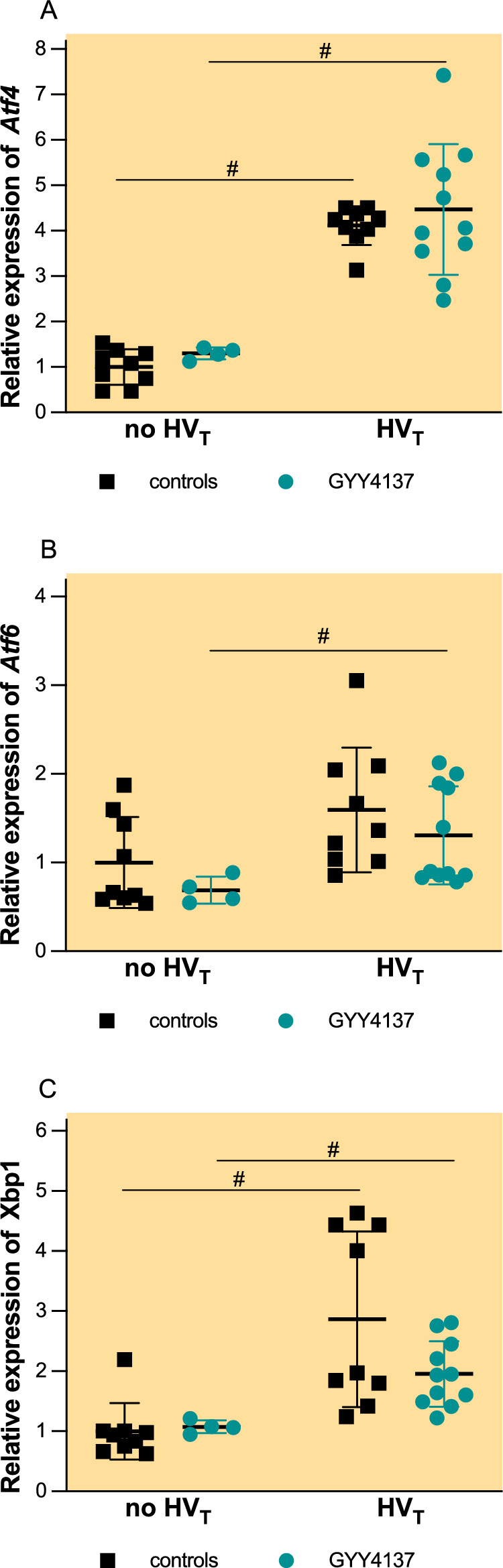
Pulmonary messenger RNA (mRNA) concentrations of *Atf4* (**A**), *Atf6* (**B**) and *Xbp1* (**C**). Groups: controls no HV_T_ (*n* = 9)—pretreatment with PBS and no mechanical ventilation, GYY4137 no HV_T_ (*n* = 4)—pretreatment with GYY4137 an no mechanical ventilation, controls HV_T_ (*n* = 9) pretreatment with PBS and HV_T_ ventilation, GYY4137 HV_T_ (*n* = 11)—pretreatment with GYY4137 and HV_T_ ventilation. mRNA concentrations are expressed as fold change relative to the average normalised expression values in no HV_T_ controls (mean ± SD). One extreme outlier from the GYY4137 no HV_T_ group was excluded from the statistical analysis. #*p* < 0.05 no HV_T_ vs. HV_T_ (Mann–Whitney *U* test)

At baseline, pretreatment with GYY4137 had no effect on pulmonary expression of *Atf4, Atf6 and Xbp1* (Fig. 7)*.*

HV_T_ ventilation resulted in a 4.1-fold increase in pulmonary *Atf4* expression (Fig. 7A, p < 0.001) and 2.8-fold increase in pulmonary *Xbp1* expression (Fig. 7C, p < 0.001) and an insignificant increase in pulmonary *Atf6* expression after HV_T_ ventilation (Fig. 7B, 1.6 ± 1, *p* = 0.062).

Pretreatment with GYY4137 did not affect the pulmonary expression of *Atf4, Atf6* and *Xbp1* in mice subjected to HV_T_ ventilation (Fig. 7).

### Hemodynamic and ventilatory parameters during HV_T_ ventilation

**Fig. 8:**
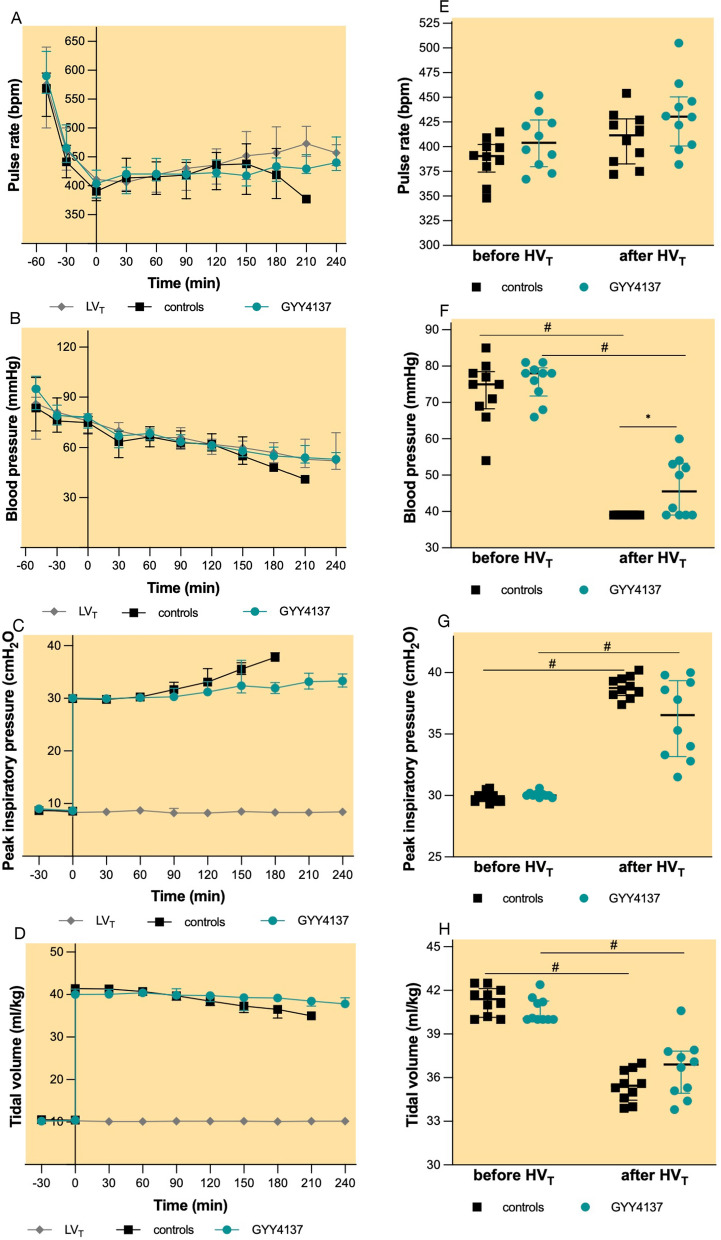
Haemodynamic and ventilatory parameters.** A** Pulse rate (bpm),** B** mean arterial blood pressure (mmHg),** C** peak inspiratory pressure (cmH_2_O) and** D** tidal volume (ml/kg) at 30 min intervals during mechanical ventilation.** E** pulse rate (bpm),** F** mean arterial blood pressure (mmHg),** G** peak inspiratory pressure (cmH_2_O) and** H** tidal volume (ml/kg) at the beginning and at the end of HV_T_ ventilation. Groups: controls (*n* = 10)—pretreatment with PBS and HV_T_ ventilation, GYY4137 (*n* = 10)—pretreatment with GYY4137 and HV_T_ ventilation, LV_T_, (* n*= 7)—pretreatment with PBS and LV_T_ ventilation. T0—start of HV_T_ ventilation. During HV_T_ ventilation mice gradually dropped out from the analysis as they died. All values are depicted as medians and IQR.^#^p < 0.05 before HVT vs. after HVT (Mann–Whitney* U* test)

**Fig. 9:**
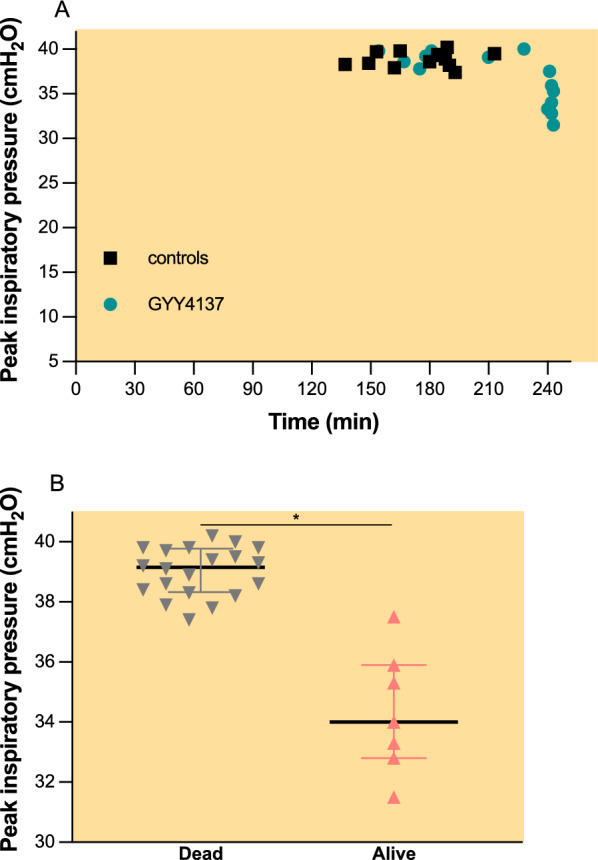
Peak inspiratory pressure at the end of experiment.** A** Scatter plot depicting peak inspiratory pressure (cmH_2_O) vs. duration of HV_T_ ventilation; controls (*n* = 13), GYY4137 (*n* = 14). ** B** Mice dead (*n* = 20) vs. alive (*n* = 7) at 240 min of HV_T_ ventilation irrespective of pretreatment. Values presented as median an IQR. *p < 0.001 Alive vs. Dead (Mann–Whitney * U* test)

At the beginning of HV_T_ ventilation, mean arterial blood pressure, pulse rate, peak inspiratory pressure, and tidal volume were similar between groups (Fig. 8). During HV_T_ ventilation, blood pressure (Fig. 8B, F) and tidal volume (Fig. 8D, H) gradually decreased, while peak inspiratory pressure increased (Fig. 8C, G). Pulse rate remained relatively constant throughout the experiment (Fig. 8A, E). Animals that did not survive the 240 min of HV_T_ ventilation had a higher peak inspiratory pressure at the time of death compared with those that completed the 240 min of HV_T_ ventilation (Fig. 9, 39 cmH_2_O, IQR 38–40 cmH_2_O vs. 34 cmH_2_O, IQR 33–36 cmH_2_O, *p* < 0.001).

## Discussion

In this study, we showed that treatment with GYY4137, a slow-releasing sulphide donor, prior to injurious HV_T_ ventilation improved survival and arterial blood oxygenation in mice. It also reduced the development of inflammation and pulmonary oedema. This protective effect of GYY4137 was not associated with the inhibition of the NFκB pathway or the endoplasmic reticulum stress response. Pretreatment with GYY4137 had little effect on Nrf2-dependent gene expression.

This is the first study to show that pretreatment with GYY4137 provides protection against VILI by reducing inflammation, as evidenced by significantly reduced levels of the pro-inflammatory cytokine IL-1β in the lungs. Interestingly, these anti-inflammatory properties were not associated with the canonical pathway of NFκB activity. NFκB is a family of inducible transcription factors involved in the inflammasome activation and pro-inflammatory cytokines production [18]. The role of sulphide species in modulating NFκB activity has been demonstrated in both in vivo and in vitro studies: in a mouse model of hyperoxia induced lung injury, pretreatment with sodium hydro-sulphide significantly reduced the canonical pathway of NFκB activity, as evidenced by reduced nuclear p65 activity [13]. In human alveolar type II-like epithelial cells infected with respiratory syncytial virus, GYY4137 reduced virus-induced activation of the canonical NFκB pathway [11]. In a more recent study, GYY4137 suppressed nuclear p65 activity in a RAW264.7 macrophage cell culture [19]. In sepsis-induced acute lung injury induced by cecal ligation and puncture, GYY4137 reduced inflammation and the activation of the canonical pathway of NFκB [20]. Despite this evidence, it remains uncertain whether the anti-inflammatory effect of GYY4137 is solely mediated via NFκB, suggesting the potential involvement of alternative inflammatory pathways [21].

Injurious HV_T_ ventilation significantly increased total protein concentration in BALF, indicating the development of lung oedema, which was associated with a reduction in respiratory system compliance. Pretreatment with GYY4137 reduced the development of lung oedema and slightly improved respiratory system compliance. These findings are consistent with a study showing that pretreatment with sodium sulphide provided similar protection against VILI [6]. In addition, GYY4137 has been shown to reduce the disruption of endothelial barrier permeability in a cell culture model infected with SARS-CoV-2 virus [22].

Previous studies have shown that H_2_S induces Nrf2 dependent gene expression [6]. Nrf2 is a transcription factor that regulates several antioxidant genes involved in the production and metabolism of antioxidant proteins, including superoxide dismutase and glutathione. In our study, pretreatment with GYY4137 significantly increased the expression of *Nqo1* and reduced the expression of *Hmox1* at baseline. Our previous work has shown that up-regulation of Nrf2-dependent gene expression prior to injurious mechanical ventilation is associated with protection against VILI [23].

*Nqo1/*NQO1 encodes NAD(P)H quinone oxidoreductase 1, which prevents the production of ROS/RNS by reducing quinones to hydroquinones. Evidence that *Nqo1* is protective in acute lung injury remains inconclusive. Upregulation of *Nqo1* has been shown to protect against hyperoxia-induced lung injury and VILI [6, 24, 25]. Conversely, a human genetic polymorphism that reduces the transcription of NQO1 provided protection against acute lung injury in patients following major trauma [26]. The authors argued that although NQO1 is generally considered to be an antioxidant, it may act as a pro-oxidant under certain conditions.

Similarly, *Hmox1*/HMOX1 encoding heme oxygenase 1 is considered protective in various models of acute lung injury. In LPS-induced lung injury, activation of *Hmox1* suppresses the level of NLRP3 inflammasomes and the subsequent increase in IL-1β levels [27]. Nrf2-dependent up-regulation of HMOX1 in pulmonary epithelial cells has been shown to protect against ferroptosis—a non-apoptotic form of cell death characterised by extensive iron-dependent lipid peroxidation [28]. In another study investigating sepsis-induced acute lung injury after cecal ligation and puncture, GYY4137 attenuated sepsis-induced ferroptosis [29]. GYY4137 also increased *Hmox1* expression in LPS-induced lung injury, which was associated with protection against lung injury [19]. It appears that heme oxygenase 1 has a more complex role in ferroptosis than previously thought. Under certain conditions, the chemical reaction catalysed by heme oxygenase 1 may exacerbate oxidative stress and facilitate ferroptosis [30].

HV_T_ ventilation itself induced a significant increase in all measured Nrf2-dependent genes (*Nrf2, Nqo1, Gpx2, Sod1, Sod2, Hmox1*), but pretreatment with GYY4137 showed no additional effect on the pulmonary expression of these genes. It is possible that the stimulus of extremely high tidal volumes may have masked any potential benefit of GYY4137. Our data are in contrast with the results of a previous study in a similar mouse model of VILI, showing that pretreatment with intra-peritoneal sodium sulphide increased the expression of *Nqo1* and *Gpx2* after HV_T_ ventilation [6]. Also, in primary human small airway epithelial cells infected with respiratory syncytial virus, GYY4137 was able to restore Nrf2-dependent gene expression of numerous Nrf2-dependent genes including *Nqo1* and *Sod1* [31]. We propose that the severity of lung injury in our model resulted in maximal up-regulation of Nrf2-dependent pathways, masking additional effects of GYY4137.

Mice deficient in cystathionine-γ-lyase (CSE)—one of the enzymes responsible for endogenous H_2_S production—develop alveolar wall thickening, diffuse interstitial oedema and leukocyte infiltration in lung tissues [32]. *Cse*-deficient mice had higher levels of pro-inflammatory cytokines and developed oxidative stress, characterised by increased levels of malondialdehyde (MDA) as well as decreased levels of superoxide dismutase and reduced GSH/GSSG. In another study, GYY4137 treatment increased superoxide dismutase activity, reversed endotoxin-induced oxidative/nitrative stress and increased the antioxidant biomarker ratio of reduced/oxidized glutathione (GSH/GSSG) [8]. However, in our study, GYY4137 did not alter the transcription of *Sod1* or *Sod2* nor did it further enhance the increase in glutathione levels induced by HV_T_ ventilation. These results contrast with a study, showing that pretreatment with sodium sulphide restored total glutathione levels and GSH/GSSG ratio in mice subjected to HV_T_ ventilation [6].

In our model, HV_T_ ventilation induced an endoplasmic reticulum (ER) stress response, as indicated by the up-regulation of *Atf4* and *Xbp1f* and, to a lesser extent, *Atf6*. These findings are in line with previous research showing the up-regulation of *Atf4* in VILI [33]. In contrast to a study showing that exogenous H_2_S suppresses ER stress markers and *Atf6* activation, GYY4137 did not attenuate these responses in our model [34].

Deterioration in lung function was evidenced by increasing peak inspiratory pressure and decreasing tidal volume, likely reflecting the development of oedema approximately 2 h after HV_T_ ventilation. This deterioration in lung function was associated with a gradual decrease in mean arterial pressure. It is likely, that the increase in inspiratory pressure affected right heart function due to intra-thoracic heart–lung interactions, contributing to mortality. The relatively stable pulse rate throughout the experiment argues against vasoplegic shock from biotrauma as the sole cause of mortality. We excluded the presence of tension pneumothorax as a possible cause of death at the end of the experiment.

This study has several limitations. First, our model induced a very severe lung injury resulting in extremely poor respiratory system compliance which may have contributed to mortality and reduced the role of biotrauma in mortality. Second, the lack of measurements of systemic inflammation limits conclusions about the role of biotrauma in mortality. Third, it is possible that the severity of the lung injury was so extreme that it activated both pro-inflammatory and protective pathways to the physiological maximum, masking any potential benefit of GYY4137.

In addition, to cause lethal lung injury, we had to use much higher tidal volumes than are typically used in the clinical practice. However, the respiratory system mechanics in mice and humans are not directly comparable. Moreover, patients with the acute respiratory distress syndrome (ARDS) experience extreme over-distension even when ventilated with small tidal volumes. Small functional lung volumes (the “baby lung” concept) and severe lung heterogeneity make ARDS patients highly susceptible to VILI [35, 36]. Therefore, pharmacological therapies such as GYY4137 that reduce lung biotrauma may complement protective ventilatory strategies to reduce the impact of VILI-associated mortality in ARDS patients.

## Conclusions

This is the first study to demonstrate that slow releasing sulphide donor GYY4137 provides protection against ventilator-induced lung injury. These findings further confirm the anti-inflammatory properties of sulphide species and highlight their therapeutic potential. Further studies are required to elucidate their mechanism of action, to differentiate the specific cell types involved in these mechanisms, and to evaluate their therapeutic efficacy in large animal models.

## Data Availability

The data sets generated and/or analysed during the current study are available in the Mendeley Data repository, data.mendeley.com/preview/nrp54 h69c2?a=c4604309-7022-49ab-bd60-fcd293 d9e808.

## Abbreviations

ARDS: Acute respiratory distress syndrome
*Atf4*: Activating transcription factor 4
*Atf6*: Activating transcription factor 6
BALF: Broncho-alveolar lavage fluid
ER: Endoplasmic reticulum
GYY4137: Morpholin-4-ium 4 methoxyphenyl(morpholino) phosphonodithioate
*Gpx2*: Glutathione peroxidase 2
GSH: Oxidized glutathione
GSSG: Reduced glutathione
*Hmox1*: Heme oxygenase 1
H_2_S: Hydrogen sulphide
HV_T_: High tidal volume
IL–1β: Interleukin 1 beta
IQR: Interquartile range
LPS: Lipopolysaccharide
LV_T_: Low tidal volume
NF-κB: Nuclear factor kappa B pathway
*Nqo1*: NAD(P)H quinone oxidoreductase
Nrf2: Nuclear erythroid 2-related factor 2
PBS: Phosphate buffered saline
PEEP: Positive end-expiratory pressure
SD: Standard deviation
*Sod1*: Superoxide dismutase 1
*Sod2*: Superoxide dismutase 2
VILI: Ventilator induced lung injury
*Xbp1*: X-box binding protein 1
